# The effects of post-hypnotic suggestion on muscular performance: an EMG study on the forearm during a static handgrip endurance test

**DOI:** 10.5114/biolsport.2025.147013

**Published:** 2025-03-24

**Authors:** Andrea De Giorgio, Taian Vieira, Cosme F. Buzzachera, Goran Kuvačić, Stefano dell’Anna, Nicola Luigi Bragazzi, Sonia Angilletta, Marco Alessandria

**Affiliations:** 1 Department of Theoretical and Applied Sciences, eCampus University; Novedrate, Como (Italy); 2 Laboratory for Engineering of the Neuromuscular System, Department of Electronics and Telecommunications, Politecnico di Torino, Turin, Italy; 3 PoliToBIOMed Lab, Politecnico di Torino, Torino, Italy; 4 Department of Public Health, Experimental and Forensic Medicine, University of Pavia, Pavia, Italy; 5 Faculty of Kinesiology, University of Split, Split, Croatia; 6 Laboratory for Industrial and Applied Mathematics, Department of Mathematics and Statistics, Faculty of Science, York University, Toronto, ON, Canada; 7 A.S.D. “SportTiVà?”, Turin, Italy; 8 Department of Life Sciences and Systems Biology, University of Turin, Turin, Italy

**Keywords:** Post-Hypnotic suggestion, Motivational suggestions, Electromyography, Imagery, Susceptibility

## Abstract

Hypnosis is known for its effects on various psychophysiological phenomena, such as perception, emotions, fatigue, and muscle strength. Besides the conflicting evidence on the influence of hypnosis on muscle performance, its role in influencing central or peripheral fatigue remains poorly understood. Here, we investigated the effects of a single hypnosis session, terminated with a precise posthypnotic suggestion, on muscle strength, endurance, and myoelectric activity. Thirty participants (M = 17, F = 13) were divided into a Control (CO) and a Hypnosis group (HY). Handgrip strength and endurance were tested in three pre- and post-training phases: i) holding the handgrip as strongly as possible for 5 seconds (i.e. a measure of muscle strength); ii) after a 1-minute passive pause, holding the handgrip as strongly and as long as possible (i.e. a measure of muscle endurance); iii) after a further 1-minute pause, the first trial was repeated. All these procedures were repeated after a 30-minute rest period during which the CO could use the time freely, the HY was subjected to the hypnosis session. During the experimental procedures, surface EMG was applied to the forearm muscles to assess neuromuscular fatigue. Regardless of a stronger improvement between pre- and post-processing in the HY, we found no interaction effect between the groups. This suggests that a single post-hypnotic suggestion is not sufficient to significantly increase the force exerted over time (i.e., impulse), and that the observed HY improvement may be influenced by highly susceptible participants. Furthermore, despite this difference, we found no change in forearm muscle activation. Our results show that a single hypnosis session negligibly altered muscular performance. These findings contribute to the debate on the topic of hypnosis and fatigue but require further investigation, given the observed tendency of the hypnosis group to delay fatigue.

## INTRODUCTION

Hypnosis can be described as a modified state of consciousness characterized by increased receptivity to suggestions aimed at altering perception [1], emotions [2, 3], fatigue [4, 5], pain [6], and muscle strength [7], among others. Hypnosis has also been used to enhance the effects of mental imagery [8], to alleviate the psychological discomfort associated with injury [9], and to improve performance in sports such as archery, basketball, and golf, when combined with relaxation [10–12]. Regarding muscle strength, as early as 1961, Ikai and Steinhaus [13] reported an average 25% increase in strength in maximal voluntary contractions (MVC) of the forearm flexor muscles after hypnotic suggestion. Mazini Filho and coworkers [7] investigated the effects of hypnotic induction on muscle strength in men with experience in resistance training. These authors showed that hypnotic induction significantly increased strength (approximately 14.8%) in the one-repetition maximum test compared to controls. However, these data are in contrast to other scientific articles. London and Fuhrer [14], for example, showed that grip strength and endurance when holding weights did not differ significantly under hypnotic and awake conditions, whereby the former condition was examined in isolation, i.e. without motivating suggestions. A few years later, Barber and Calverley [15] found that grip strength did not differ between hypnotic and awake experimental treatments without motivational suggestions. More recently, a study utilizing transcranial magnetic stimulation (TMS) of the primary motor area of the right hand compared different types of hypnosis. The results revealed a significant increase in cortical excitability and endurance tolerance at 30% of the MVC, but only in the task-motivational hypnosis group. This effect was not observed in the conventional hypnotic conditioning or task-motivational groups [16]. Interestingly, a similar study on knee extensors showed no differences in MVC, time-to-task failure, cortical excitability, and voluntary activation between control and hypnosis conditions in a cross-sectional design study [17]. Regarding neurophysiological parameters, Williamson and colleagues [18] investigated brain activation during real and imaginary handgrip under hypnosis. The authors showed that hypnosis affects the activation of central control, highlighting a similar activation of brain areas during imagined and real exercises. In addition, electromyography (EMG) data indicated altered neuromuscular activation under hypnosis, providing insight into the effects on central fatigue. On the other hand, hypnosis has been shown to reduce EMG amplitude in the masseter, temporalis, and frontalis muscles [19–21], but some authors concluded that the actual effect of hypnosis could not be confirmed due to intrinsic EMG limitations [21]. Furthermore, in their study, Peter and collaborators [22] investigated the strain and muscle activity during lifting and holding up the right arm for 3 minutes. During hypnotic arm lifting, total muscle activity was significantly lower compared to voluntary holding, especially in terms of deltoid muscle activity. Without hypnosis, muscle activity correlated positively with the load, while no such correlation was found under hypnosis. While this article was being written, a similar study to ours was published, though without the use of EMG, reaching the same result [23]. Both hypnosis and control group showed no significant differences in handgrip objective strength immediately afterward; however, significant differences emerged one week after the experimental session. Based on what has been introduced so far and in light of the recent literature [24] it is possible to say that the hypnotic effect on muscle strength and muscle endurance is not yet fully elucidated.

In this study, we aimed to investigate whether a single hypnotic induction can alter EMG parameters, MVC, and forearm muscle endurance using a handgrip strength test. Our hypothesis was that hypnosis can increase muscle strength, delay fatigue-induced myoelectric changes, and improve muscle endurance, even in the absence of motivational suggestions. A better understanding of the effectiveness of hypnotic manipulations on parameters of neuromuscular function that could ultimately affect athletic performance could be of benefit to sports medicine practitioners and sports psychologists.

## MATERIALS AND METHODS

### Participants

Participants were recruited from psychotherapy schools, postgraduate schools of hypnosis, and extended acquaintances and participated voluntarily in this study. A total of 30 participants (F = 13; M = 17; mean age: 42.33; SD: 15.87) were recruited and divided into Control (CO) and Hypnosis (HY) groups. In order to detect an effect of partial η^2^ of 0.14 [25] with an α level of 0.05 and a power of 0.80 in a two-way ANOVA with repeated measures and a nonsphericity correction equal to 1, 28 participants would be required (G*Power; 3.1.9.6 version).

The inclusion criteria were age > 18 years; no previous experience with hypnosis; no neurological diseases; no psychiatric disorders (including schizophrenia, psychosis, and borderline personality disorder); no cardiac diseases (especially hypertension); no respiratory diseases; no musculoskeletal diseases in the upper limbs, shoulders, and neck; and no intense physical activity on the previous day. We also asked the participants not to drink coffee or alcohol from 2 p.m. the day before and not to smoke from the morning of the trial session. The study protocol was conducted in accordance with the Declaration of Helsinki and approved by the research ethics committees of the University of Split (Protocol 2181-205-02-05-24-0027 approved March 1, 2024). Prior to participation, each participant gave written, informed consent.

### Procedures

**FIG. 1:**
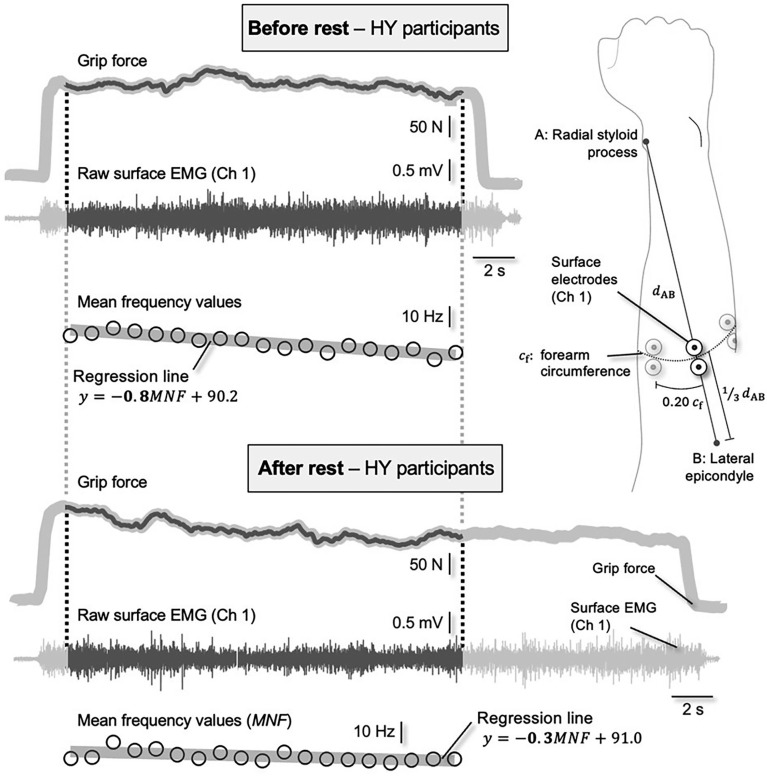
Example of raw data (grip force and surface EMGs) and processed data (mean frequency) of a representative participant in HY. The EMGs were collected considering the relative electrode position shown in the schematic illustration. EMGs were processed over the same duration corresponding to the shortest endurance test. The regression lines were calculated for the MNF values by minimizing the mean square residuals. The slope of the regression lines (bolded in the equations) obtained for each EMG channel was used to assess how much, where and how extensively myoelectric fatigue manifested in the forearm muscles.

During the first visit to the laboratory, the susceptibility of all participants to hypnotic suggestions was assessed using Form C of the Stanford Hypnotic Susceptibility Scale [25]. Receptivity to hypnosis has been shown to be related to positive responses to at least three of the following four indicators: closing the eyes, lowering the right hand, rigidity of the right arm, and bringing the hands together [26]. Participants who scored ≥ 3 of the 4 indicators were included in HY, while those who scored less than 3 were assigned to CO. All procedures were performed by a psychologist who was a hypnotherapist specializing in traditional and rapid hypnotic induction techniques with ten years of experience. Upon arrival at the laboratory, participants were seated in a comfortable position for 10 minutes. They were familiarized with the study procedures, including the application of the handgrip, and the electrodes were attached to the participants (see EMG detection section; Fig. 1). The handgrip test was, then, performed. In brief, participants were asked to bend their elbow to 90 degrees and move closer until their torso touched the table and the forearm rested. The arm was taped to the torso and restrained in such a way as to prevent compensatory movement. At this point, they were instructed to i) hold the handgrip as strongly as possible for 5 seconds (i.e. a measure of muscle strength); ii) after a 1-minute passive pause, hold the grip as strongly and as long as possible (i.e. a measure of muscle endurance); iii) after a further 1-minute pause, the first trial was repeated. All these procedures were repeated after a 30-minute rest period. All experiments were performed at the same time of day to avoid circadian variations and in a room with controlled temperature (21°C; Table 1). Although both the CO and HY groups underwent the same procedures, participants in the HY group received a single hypnosis session during the 30-minute rest period between trials while participants in the CO group freely employed the same time. Once the participant was in a trance, the hypnotist gave the following post-hypnotic suggestion: “*Every time you pick up the handgrip again and feel its weight, temperature, and texture, you will immediately feel a surge of energy in your forearm and hand. The more energy you feel, the tighter you will hold the handgrip. And the tighter you hold it, the more energy you will feel in your hand, forearm, and entire body. This energy will give you even more strength and energy to hold*”. At the end of the induction, the hypnotist returned the participant to a normal waking state, accompanied him to perform the second test and then left the room without giving any further suggestions.

**TABLE 1:** Phases of the study: T0 – T1: 1 month; T1 – T2: 1 week; T3 – T4 – T5: 2 months; T6 – T7: 1 week; CO: control group; HY: hypnosis group; SHSS: Stanford Hypnotic Susceptibility Scale.

	T0	T1	T2	T3 – Before		T4 – Between trials		T5 – After		T6	T7

				CO	HY	CO	HY	CO	HY		
**Recruitment**	X	X									

**Eligibility Criteria**		X	X								

**Informed Consent**			X								

**SHSS**				X	X						

**Familiarization study procedures**				X	X						

**Trials/Data Collection**											

Electrode position				X	X						

Handgrip test/EMG				X	X			X	X		

Intervention						V (1)	V (2)				

**Statistical Analysis**										X	X

(1) Freely employed time; (2) Hypnosis session with post-hypnotic suggestion;

### Measures

#### Muscle strength and muscle endurance

Handgrip force was assessed with a pre-calibrated isometric strain gauge (MLT004/ST Grip Force Transducer, ADInstruments, New Zealand) connected to an analog-to-digital data-acquisition system (PL3516 PowerLab 16/35, ADInstruments, New Zealand) at a 1000 Hz sampling frequency (LabChart 8.1.30; ADInstruments, New Zealand).

### EMG detection

Bipolar surface EMGs were recorded from the adjacent forearm muscles during the handgrip test. Five pairs of circular electrodes (24 mm diameter, Kendall™ ECG, H124SG) were used, with the electrodes in each pair aligned longitudinally along the forearm with a center-to-center distance of 3 cm [27]. Although this number may be relatively high for the small forearm muscles, we wanted to minimize type II error [28] and ensure that most forearm muscles were represented in the surface EMGs. The center of each pair of electrodes was placed along a circumference located one-third of the distance between the styloid processes of the forearm. This circumference was determined by dividing the distance between the styloid process of the radius and the lateral epicondyle of the humerus by three. The distance between consecutive pairs of electrodes was set at 20% of this circumference (Fig. 1). The EMGs were sampled with a 16-bit A/D converter at 2 kHz (amplifier with a bandwidth amplifier of 10–500 Hz, Cometa Systems, Italy).

### Data Analysis

#### Assessing myoelectric manifestations of fatigue

All raw EMGs were visually inspected for power line interference and contact problems: none were discarded. Then, EMGs were trimmed from 1 s after the force was increased to the MVC level to 1 s before failure, when the force dropped below the noise level. To ensure similar comparisons, EMGs collected both before and after the intervention (rest in the CO) were truncated to the shortest duration before and after the intervention. For each channel, the EMG power spectrum for 1 s segments was calculated by squaring the absolute value of the Fourier transform of the raw signal. This number provides a sufficiently high spectral resolution (1 Hz) while allowing the assessment of spectral changes during the course of a fatiguing task [29]. The mean power frequency (MNF) was then computed by weighting and averaging the spectral frequencies. As the MNF is sensitive to changes in muscle fiber conduction velocity [30], it has been successfully used to assess the myoelectric manifestations of fatigue during both isometric [29] and dynamic contractions [31]. Accordingly, we calculated here the slope of the regression lines calculated via MNF and considered this slope (Fig. 1) as a proxy for myoelectric fatigue during the pre- and post-trials. For each subject and condition, we obtained five slope values, one for each channel. Three descriptors were considered to evaluate the effect of hypnosis on the myoelectric manifestation of fatigue. First, we segmented the channels that provided MNF slopes whose absolute value was greater than 70% [32] of the maximum absolute slope among the channels. Therefore, we calculated: i) the mean MNF value across the segmented channels; ii) the number of segmented channels; and iii) the centroid of the segmented channels, defined as the weighted average of the coordinates at which surface EMGs were collected from the forearm. These descriptors each indicate how strong, how extensive and where in the forearm the myoelectric fatigue can occur.

### Assessing surrogates of muscle function

Endurance and MVC trials were evaluated separately. How strongly the participants held to the handgrip during the MVCs was assessed by averaging the force value over 1 s windows, centered within the 5 s MVC trial. Four 100% MVC force values were obtained for each participant, two (before and after) the first trial session and two (before and after) the second trial session. Muscle endurance was also assessed separately for each experimental session. The fluctuation in muscle strength, computed as the standard deviation of strength, was taken into account to assess how consistently participants were able to maintain MVC levels during the endurance test. To account for how strongly and for how long participants held onto the handle, we numerically integrated the force over time, from the first moment the force increased to the first moment it dropped back to the noise level. The impulse values before and after rest (CO) and hypnosis (HY) were calculated using the trapezoidal rule for integration.

### Statistical analysis

The t-test or the U Mann-Whitney test was used to test for significant differences and to identify possible confounding factors after the distribution of the variables age, weight, height, and BMI of the two groups had been controlled using the Shapiro-Wilk normality test. Fisher’s exact test was used to check the equal distribution of gender between the groups. A two-way ANOVA with repeated measures was performed to analyze the effect of hypnosis on the EMG, MVC, and endurance variables after the normal distribution of the dependent variables was established. The factors considered were the “time” as within subjects (two levels: baseline and after) and the “groups” as between subjects (two levels: CO and HY). In addition, for all variables where the distribution was skewed, the U Mann–Whitney test was used to compare the groups at baseline and post-intervention, and the Wilcoxon test was used to compare the groups at baseline and post-intervention. Finally, a linear mixed model (LMM) was used to test for significant differences in the variable “failure time from start”. In this model, we used the average impulse rate as a covariate because the force developed over time was heterogeneous among all participants. After comparing the estimated Akaike information criterion, a random intercept and unstructured matrix were modeled. The model included fixed effects for the groups, time, and impulse rate, with a random intercept for participants at two time points. The model coefficients were estimated using maximum likelihood estimation. The likelihood ratio test was performed to test the goodness of fit between the model with and without covariates. The Bonferroni correction for multiple comparisons was applied and α was set to 0.05. IBM SPSS Statistic 29.0.1.0 was used to analyze the data.

## RESULTS

### Demographic characteristics

No significant difference was found between the groups in terms of age, weight, BMI variables and gender distribution. The variable height showed a significant difference between the groups (t28 = -2.667; p = .013). The results – along with sociodemographic characteristics – are summarized in Table 2.

**TABLE 2:** Demographic characteristics of the groups involved in the study.

	Hypnosis (n = 15)	Control (n = 15)	Comparison between groups
**Gender**	6M – 9F	11M – 4F	p = .139
**Age (years), (IQR)**	48 (34)	43 (34)	p = .285
**Weight (kg), Means (SD)**	67.40 (17.99)	71.73 (15.79)	p = .489
**Height (m), Means (SD)**	1.67 (12.06)	1.77 (10.18)	p = .013*
**BMI, Means (SD)**	24.11 (5.19)	22.54 (3.30)	p = .331

IQR, interquartile range; SD, Standard deviation; BMI, body mass index; p, p-value. M, Male. F, Female;

* significant value (p < .05).

### EMG variables

The non-parametric analysis revealed no significant differences in the mean MNF slope (U Mann-Whitney test at baseline: p = .345; U Mann-Whitney test after the intervention: p = .412; Wilcoxon test CO: p = .233; Wilcoxon test HY: p = .609) and on the MNF NCHS (U Mann-Whitney test at baseline: p = .870; U Mann-Whitney test post-intervention: p = .838; Wilcoxon test CO: p = .952; Wilcoxon test HY: p = .744). For the MNF focus, the ANOVA revealed no significant differences (main effect within subjects: F_1,28_ = .116, p = .736; between-subjects effect: F_1,28_ = 2.032, p = .165; interaction time × groups: F_1,28_ = .595, p = .447; pairwise comparison at baseline: p = .593).

### MVC variables

ANOVA revealed no significant differences in MVC strength, both pre-intervention (main effect within subjects: F_1,28_ = .526, p = .474; between-subjects effect: F_1,28_ = 3.900, p = .058; time × group interaction: F_1,28_ = .166, p = .687; pairwise comparison at baseline: p = .067) and after the intervention (main effect within subjects: F_1,28_ = .003, p = .957; between-subjects effect: F_1,28_ = 3.893, p = .058; interaction time × groups: F_1,28_ = .294, p = .592; pairwise comparison at baseline: p = .055).

### Endurance variable

**FIG. 2:**
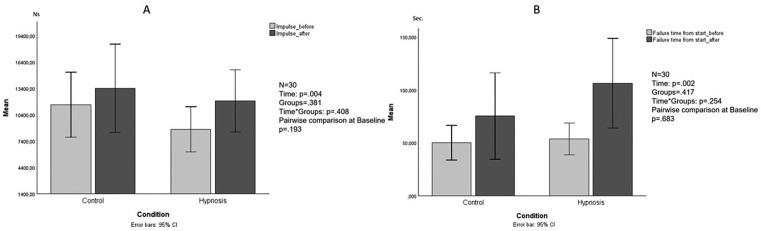
Mean values before (baseline) and after intervention impulse variable (A) and failure time (B). Ns: Newton*Second; Sec.: seconds.

The non-parametric analysis revealed no significant differences in strength fluctuation (U Mann-Whitney test at baseline: p = .089; U Mann-Whitney test after the intervention: p = .217; Wilcoxon test CO: p = .865; Wilcoxon test HY: p = .865). For the impulse variable, the ANOVA revealed significant differences (main effect within subjects: F_1,28_ = 9.725, p = .004, partial η^2^ = .258; between-subjects effect: F_1,28_ = .791, p = .381; interaction time × groups: F_1,28_ = .706, p = .408; pairwise comparison to baseline: p = .193; Figure 2A). For the variable “failure time from start” the LMM showed a significant difference in time (F_1,30.441_ = 11.887, p = .002) and impulse rate (F_1,30.582_ = 39,117, p < .001, partial η^2^ = .314), no significant difference was found between the groups (F_1,27.781_ = 1.351, p = .254) and in the interaction term time × group (F_1,30.267_ = 1.351, p = .254; Fig. 2B).

## DISCUSSION

The present study was designed to test the hypothesis that hypnotic suggestions would increase both MCV and endurance during handgrip contraction. Contrary to our hypothesis, we did not find an interaction effect between the groups. The statistical result can be explained by simple chance, but it is also possible – based on our empirical observations – to assume that it may be due to the fact that participants within the HY showed a very heterogeneous level of depth of hypnotic trance, characteristic, observed by the hypnotist through their experience. The literature that has examined MVC and hypnosis is sparse and also quite controversial. In general, a larger mean difference between before and after in the HY group compared to CO is consistent with the studies that have shown that hypnosis affects not only the perception of pain and fatigue but also performance [3, 7, 11, 31]. The fact that strength improved over time is consistent with seminal studies such as those of Ikai and Steinhaus [13], who reported an approximate 25% increase in MVC under hypnosis. However, our findings are consistent with other studies, such as that of Dittrich and coworkers [17], which found no increase in MVC for knee extensors. As mentioned in the introduction, there are some conflicting results in the existing literature regarding the effects of hypnosis on performance, particularly strength. London and Fuhrer [14], for example, found no significant differences in grip strength or endurance under hypnosis compared to awake, although the authors do not explicitly state what type of hypnosis – or posthypnotic suggestion – was performed. Although we used a specific post-hypnotic suggestion with the aim of improving both strength and endurance, conditions that seem to be crucial for maximizing the effects of hypnosis [7], we did not find significant differences between the groups. Another difference was that our HY included both men and women, whereas London and Fuhrer [14] included only women. On the other hand, Mazini Filho and coworkers [7] have shown that hypnotic induction has a positive effect on improving maximal strength in individuals by promoting a higher number of repetitions for the same load. However, in contrast to our study, the authors examined 1-RM in the upper limbs, included only male participants, and did not examine EMG. Furthermore, while this article was being written, Nieft and colleagues (2024) conducted an experiment similar to ours, but without EMG, and came to results that are consistent with ours. Participants were divided into two groups: an experimental group that received a hypnosis session and a control group that read the Schwarzenegger’s autobiography during the same period. Handgrip force was measured at four time points: before the hypnosis session, immediately after and one week later, both before and after activation of the posthypnotic suggestion. The subjective feeling of strength was measured using a visual analogue scale. The results showed that the post-hypnotic suggestion primarily increased subjective perception. Interestingly, force values increased significantly one week after the intervention, but not immediately after. This article may suggest the effects discussed in our HY alone may have led to a significant interaction between the groups after one week. This research further confirms the great heterogeneity of results in the literature about the topic strength and hypnosis. In our opinion, given the extensive experience of the hypnotist who conducted the experimental sessions, and supported by the literature, the discrepancy between the experiments is most likely due to differences in the suggestibility and hypnotic depth of the participants. In fact, it has already been discussed that depth of hypnosis and hypnotic suggestibility are associated with individual differences in neuroanatomy and/ or levels of functional connectivity [34], to the point that hypnotic depth and hypnotic suggestibility should not be considered synonyms. We would like to emphasize once again that suggestibility and depth of hypnosis are not the same thing. The former describes how easily a person can enter a hypnotic trance, while the latter indicates how deeply they enter that trance. In this context, it is important to know that the suggestibility index is not proportional to the depth of hypnosis. In other words, it is possible for a person who is less suggestible than another to reach a greater depth during hypnosis.

A strength of our research is the use of forearm EMG recording during the experimental sessions. In our experimental setup, the EMG frequency descriptor during contraction – and for the same duration – showed no differences between the groups before and after the intervention. It has been highlighted in the literature that the accumulation of metabolites in the muscle fibers, which is a consequence of the fatigue process, alters the pH and consequently the speed at which the action potential propagates along the muscle fiber [33]. Since the reduction in conduction velocity directly affects the mean frequency [34], the frequency descriptor should have changed significantly between groups, which was not the case. Therefore, the sustained voluntary activation of the muscles by the influence of hypnosis on the central neural mechanisms could explain the observed significant improvement in the impulse value, albeit only in the HY. However, the lack of interaction between the groups does not allow us to determine whether hypnosis is able to modulate central or peripheral fatigue. The notion that central fatigue can be at least partially modulated by hypnosis is supported by a study by Takarada and Nozaki [16]. The researchers used transcranial magnetic stimulation of the primary motor cortex to investigate how hypnosis, together with a concurrent suggestion that increased motivation for a strength task, affected the state of the motor system. An increase in corticospinal excitability leading to increased effort was observed, but only when the motivational suggestion was administered during the hypnotic induction. This result suggests that the hypnotic suggestion significantly altered the state of the primary motor cortex and the corresponding behavior. Taken together, these evidences seem to contradict previous studies [7, 13], as Takarada and Nozaki [16] which found no change in MVC by hypnotic suggestion alone. Regarding MVC strength and central/peripheral mechanisms, an interesting study by Ranganathan and colleagues [35] provides valuable insights into these processes. The researchers investigated whether mental imagery alone could lead to increases in strength in the little finger abductors and elbow flexors. Thirty healthy participants were divided into groups that either performed mental contractions or served as a control group, with some participants performing MVC for comparison. The results showed that mental training alone increased finger abduction strength by 35% and elbow flexion strength by 13.5%. Even more interestingly, these gains were accompanied by a significant increase in brain activity as measured by cortical potentials. The authors concluded that mental training can improve muscle strength by enhancing brain signals to the muscles and suggested that neural adaptations, rather than muscle hypertrophy, were responsible for these improvements. More recently, Dittrich and coworkers [17] investigated whether hypnosis can modulate corticospinal excitability by examining its effects on knee extensor muscles. The authors found no effect of hypnotic suggestions on corticospinal excitability and intracortical inhibition but reported a significant increase in the amplitude of the motor evoked potential (MEP) and the short-term relationship between intracortical inhibition and MEP towards the end of exercise. If an increase in MEP amplitude and the Short-Interval Intracortical Inhibition/MEP ratio is observed at the end of the exercise in the hypnosis group, this would indicate that hypnosis facilitates greater activation of the muscle groups involved. According to the existing literature, increased corticospinal excitability is typically associated with increased EMG activity, reflecting a stronger neural drive through the motor cortex [36, 37]. The observed changes thus suggest that hypnosis increased the performance of the motor cortex, leading to increased muscle recruitment during the task. However, this appears to be limited to participants who are particularly susceptible to hypnosis. In contrast to the cited authors, the hypnotherapist in our study was not involved in the hypnotic inductions during the exercise and no motivational suggestions were given, so this potential confounding factor could be excluded, and the effect of hypnosis alone could be isolated. Although these studies appear contradictory, one plausible explanation is that this effect is mediated by increased recruitment of motor units or a reduction in inhibitory signals that normally limit voluntary muscle contraction under fatiguing conditions [38, 39]. However, it is important to emphasize that by using the handgrip, we indirectly observed the behavior of smaller motor units and found an improvement in the impulse value under MVC conditions after the intervention. Considering that our results would probably have been different if we had found a group of people with the same high hypnotic susceptibility, it can be concluded that hypnosis during MVCs has a stronger effect during exercise with small motor units than during submaximal efforts with large muscle groups. This effect seems to act mainly on mechanisms related to the central nervous system, including fatigue. In addition, hypnosis could influence the top-down regulation of attentional processes, as suggested by studies in which changes in functional connectivity between the anterior cingulate cortex and neural networks involved in cognitive and emotional processing were observed [3]. These mechanisms could also partly explain the pulse increase we observed, as a reduction in central fatigue would allow hypnotized individuals to maintain more efficient muscle activation during the task, which would increase the pulse generated over time. In conclusion, our results do not fully support the literature suggesting that hypnosis can positively influence strength endurance by modulating central fatigue. Therefore, due to the limited and conflicting results in the current literature, further studies are needed that simultaneously consider central and peripheral fatigue parameters, different levels of hypnotic suggestibility, submaximal and maximal strength, and psychological profiles of participants.

## CONCLUSIONS

Several limitations must be acknowledged. The differences observed in the HY are coincidental, however, this aspect does not necessarily represent a limitation. Rather, the result could be due to the large statistical variability of the variables of interest, which in turn is caused by the heterogeneity of the depth of hypnosis of the individual participants. In addition, the hypothesis regarding central fatigue during the MVC handgrip test induced by hypnosis requires further investigation to confirm our above hypotheses. Finally, the p-values for the MVC variables are close to the significance threshold, and we hypothesize that the lack of statistical significance may be due to the limited number of hypnosis sessions. With regard to this point, it is important to emphasize that a homogeneous hypnosis group, which is particularly susceptible to hypnosis, would in all likelihood have led to clearer and more encouraging results. For this reason, a further study with specific parameters for allocation to the hypnosis group could lead to clearer results.

## References

[1] Zahedi A, Stuermer B, Hatami J, Rostami R, Sommer W. Eliminating stroop effects with post-hypnotic instructions: Brain mechanisms inferred from EEG. Neuropsychologia. 2017; 96:70–7. Epub 2017/01/13. doi: 10.1016/j.neuropsychologia.2017.01.006. PubMed PMID: 28077327. 28077327

[2] Meaues A. ANXIETY AND HYPNOSIS. Medical Journal of Australia. 1966; 1. 10.5694/j.1326-5377.1966.tb72403.x 5905385

[3] Wolf TG, Schläppi S, Benz CI, Campus G. Efficacy of Hypnosis on Dental Anxiety and Phobia: A Systematic Review and Meta-Analysis. Brain Sci. 2022; 12(5). Epub 2022/05/29. doi: 10.3390/brainsci12050521. PubMed PMID: 35624907; PubMed Central PMCID: PMCPMC. 35624907 PMC9138388

[4] Untas A, Chauveau P, Dupré-Goudable C, Kolko A, Lakdja F, Cazenave N. The effects of hypnosis on anxiety, depression, fatigue, and sleepiness in people undergoing hemodialysis: a clinical report. Int J Clin Exp Hypn. 2013; 61(4):475–83. Epub 2013/08/21. doi: 10.1080/00207144.2013.810485. PubMed PMID: 23957264. 23957264

[5] Braley TJ, Segal BM, Chervin RD. Hypnotic use and fatigue in multiple sclerosis. Sleep Med. 2015; 16(1):131–7. Epub 2014/12/03. doi: 10.1016/j.sleep.2014.09.006. PubMed PMID: 25454981. 25454981

[6] Caron-Trahan R, Jusseaux AE, Aubin M, Cardinal É, Aramideh J, Idrissi M, et al. Practicing self-hypnosis to reduce chronic pain: A qualitative exploratory study of HYlaDO. Br J Pain. 2024; 18(1):28–41. Epub 2024/02/12. doi: 10.1177/20494637231200324. PubMed PMID: 38344266; PubMed Central PMCID: PMCPMC. 38344266 PMC10851891

[7] Mazini Filho M, Savoia R, Brandão Pinto de Castro J, Moreira O, Venturini G, Curty V, et al. Effects of hypnotic induction on muscular strength in men with experience in resistance training. Journal of Exercise Physiology Online. 2018; 21:52–61.

[8] Liggett DR. Enhancing imagery through hypnosis: a performance aid for athletes. Am J Clin Hypn. 2000; 43(2):149–57. Epub 2000/10/07. doi: 10.1080/00029157.2000.10404267. PubMed PMID: 11022364. 11022364

[9] Iglesias A, Iglesias A. Clinical hypnosis with a Little League baseball population: performance enhancement and resolving traumatic experiences. Am J Clin Hypn. 2011; 53(3):183–91. Epub 2011/03/17. doi: 10.1080/00029157.2011.10401756. PubMed PMID: 21404954. 21404954

[10] Robazza C, Bortoli L. A case study of improved performance in archery using hypnosis. Percept Mot Skills. 1995; 81(3 Pt 2):1364–6. Epub 1995/12/01. doi: 10.2466/pms.1995.81.3f.1364. PubMed PMID: 8684935. 8684935

[11] Pates J, Cummings A, Maynard I. The effects of hypnosis on flow states and three-point shooting performance in basketball players. The Sport Psychologist. 2002; 16(1):34–47.

[12] Nicholls AR, Poltnan RCJ, Holt NL. The Effects of Individualized Imagery Interventions on Golf Performance and Flow States. Athletic Insight: The Online Journal of Sport Psychology. 2005; 7(1): No Pagination Specified-No Pagination Specified.

[13] Ikai M, Steinhaus AH. Some factors modifying the expression of human strength. J Appl Physiol. 1961; 16:157–63. Epub 1961/01/01. doi: 10.1152/jappl.1961.16.1.157. PubMed PMID: 13717441. 13717441

[14] London P, Fuhrer M. Hypnosis, motivation, and performance. Journal of Personality. 1961; 29(3):321–33. doi: 10.1111/j.1467-6494.1961.tb01665.x. 13763304

[15] Barber TX, Calverley DS. AN EXPERIMENTAL STUDY OF “HYPNOTIC” (AUDITORY AND VISUAL) HALLUCINATIONS. J Abnorm Psychol. 1964; 68:13–20. Epub 1964/01/01. doi: 10.1037/h0042175. PubMed PMID: 14105174. 14105174

[16] Takarada Y, Nozaki D. Maximal voluntary force strengthened by the enhancement of motor system state through barely visible priming words with reward. PLoS One. 2014; 9(10):e109422. Epub 2014/10/03. doi: 10.1371/journal.pone.0109422. PubMed PMID: 25275612; PubMed Central PMCID: PMCPMC. 25275612 PMC4183639

[17] Dittrich N, Agostino D, Antonini Philippe R, Guglielmo LGA, Place N. Effect of hypnotic suggestion on knee extensor neuromuscular properties in resting and fatigued states. PLoS One. 2018; 13(4):e0195437. Epub 2018/04/24. doi: 10.1371/journal.pone.0195437. PubMed PMID: 29684047; PubMed Central PMCID: PMCPMC. 29684047 PMC5912755

[18] Williamson JW, McColl R, Mathews D, Mitchell JH, Raven PB, Morgan WP. Brain activation by central command during actual and imagined handgrip under hypnosis. J Appl Physiol (1985). 2002; 92(3):1317–24. Epub 2002/02/14. doi: 10.1152/japplphysiol.00939.2001. PubMed PMID: 11842073. 11842073

[19] Miller LS, Cross HJ. Hypnotic susceptibility, hypnosis, and EMG biofeedback in the reduction of frontalis muscle tension. Int J Clin Exp Hypn. 1985; 33(3):258–72. Epub 1985/07/01. doi: 10.1080/00207148508406654. PubMed PMID: 4030155. 4030155

[20] Manns A, Zuazola RV, Sirhan RM, Quiroz M, Rocabado M. Relationship between the tonic elevator mandibular activity and the vertical dimension during the states of vigilance and hypnosis. Cranio. 1990; 8(2):163–70. Epub 1990/04/01. doi: 10.1080/08869634.1990.11678310. PubMed PMID: 2073696. 2073696

[21] Al-Enaizan N, Davey KJ, Lyons MF, Cadden SW. Effect of hypnosis on masseter EMG recorded during the ‘resting’ and a slightly open jaw posture. J Oral Rehabil. 2015; 42(11):840–6. Epub 2015/06/11. doi: 10.1111/joor.12316. PubMed PMID: 26059538. 26059538

[22] Peter B, Schiebler P, Piesbergen C, Hagl M. Electromyographic investigation of hypnotic arm levitation: differences between voluntary arm elevation and involuntary arm levitation. Int J Clin Exp Hypn. 2012; 60(1):88–110. Epub 2011/11/22. doi: 10.1080/00207144.2011.622213. PubMed PMID: 22098572. 22098572

[23] Nieft U, Schlütz M, Schmidt B. Increasing handgrip strength via post-hypnotic suggestions with lasting effects. Scientific Reports. 2024; 14(1):23344. doi: 10.1038/s41590-024-73117-0. 39402088 PMC11473724

[24] Zahedi A, Jay Lynn S, Sommer W. How hypnotic suggestions work – A systematic review of prominent theories of hypnosis. Conscious Cogn. 2024; 123:103730. Epub 2024/07/21. doi: 10.1016/j.concog.2024.103730. PubMed PMID: 39032268. 39032268

[25] Hilgard ER, Weitzenhoffer AM, Landes J, Moore RK. The distribution of susceptibility to hypnosis in a student population: A study using the Stanford Hypnotic Susceptibility Scale. Psychological Monographs: General and Applied. 1961; 75(8):1–22. doi: 10.1037/h0093802.

[26] Moran TE, Kurtz RM, Strube MJ. The efficacy of the Waterloo-Stanford Group Scale of hypnotic susceptibility: form C. Am J Clin Hypn. 2002; 44(3–4):221–30. Epub 2002/01/22. doi: 10.1080/00029157.2002.10403482. PubMed PMID: 11799536. 11799536

[27] Vieira TM, Cerone GL, Botter A, Watanabe K, Vigotsky AD. The Sensitivity of Bipolar Electromyograms to Muscle Excitation Scales With the Inter-Electrode Distance. IEEE Trans Neural Syst Rehabil Eng. 2023; 31:4245–55. Epub 2023/10/16. doi: 10.1109/tnsre.2023.3325132. PubMed PMID: 37844006. 37844006

[28] Vieira TM, Botter A. The Accurate Assessment of Muscle Excitation Requires the Detection of Multiple Surface Electromyograms. Exerc Sport Sci Rev. 2021; 49(1):23–34. Epub 2020/10/13. doi: 10.1249/jes.0000000000000240. PubMed PMID: 33044329. 33044329

[29] Vieira TM, Cerone GL, Bruno M, Bachero-Mena B. Myoelectric manifestations of fatigue of the finger flexor muscles and endurance capacity in experienced versus intermediate climbers during suspension tasks. J Sports Sci. 2024; 42(8):655–64. Epub 2024/05/25. doi: 10.1080/02640414.2024.2357470. PubMed PMID: 38794799. 38794799

[30] Arendt-Nielsen L, Mills KR. The relationship between mean power frequency of the EMG spectrum and muscle fibre conduction velocity. Electroencephalogr Clin Neurophysiol. 1985; 60(2):130–4. Epub 1985/02/01. doi: 10.1016/0013-4694(85)90019-7. PubMed PMID: 2578364. 2578364

[31] Bonato P, Roy SH, Knaflitz M, De Luca CJ. Time-frequency parameters of the surface myoelectric signal for assessing muscle fatigue during cyclic dynamic contractions. IEEE Trans Biomed Eng. 2001; 48(7):745–53. Epub 2001/07/10. doi: 10.1109/10.930899. PubMed PMID: 11442286. 11442286

[32] Vieira TM, Merletti R, Mesin L. Automatic segmentation of surface EMG images: Improving the estimation of neuromuscular activity. J Biomech. 2010; 43(11):2149–58. Epub 2010/05/07. doi: 10.1016/j.jbiomech.2010.03.049. PubMed PMID: 20444452. 20444452

[33] Tornero-Aguilera JF, Jimenez-Morcillo J, Rubio-Zarapuz A, Clemente-Suárez VJ. Central and Peripheral Fatigue in Physical Exercise Explained: A Narrative Review. Int J Environ Res Public Health. 2022; 19(7). Epub 2022/04/13. doi: 10.3390/ijerph19073909. PubMed PMID: 35409591; PubMed Central PMCID: PMCPMC. 35409591 PMC8997532

[34] Brody LR, Pollock MT, Roy SH, De Luca CJ, Celli B. pH-induced effects on median frequency and conduction velocity of the myoelectric signal. J Appl Physiol (1985). 1991; 71(5):1878–85. Epub 1991/11/01. doi: 10.1152/jappl.1991.71.5.1878. PubMed PMID: 1761486. 1761486

[35] Ranganathan VK, Siemionow V, Liu JZ, Sahgal V, Yue GH. From mental power to muscle power--gaining strength by using the mind. Neuropsychologia. 2004; 42(7):944–56. Epub 2004/03/05. doi: 10.1016/j.neuropsychologia.2003.11.018. PubMed PMID: 14998709. 14998709

[36] Devanne H, Lavoie BA, Capaday C. Input-output properties and gain changes in the human corticospinal pathway. Exp Brain Res. 1997; 114(2):329–38. Epub 1997/04/01. doi: 10.1007/pl00005641. PubMed PMID: 9166922. 9166922

[37] Taylor JL, Gandevia SC. Transcranial magnetic stimulation and human muscle fatigue. Muscle Nerve. 2001; 24(1):18–29. Epub 2001/01/11. doi: 10.1002/1097-4598(200101)24:1 < 18::aid-mus2 > 3.0.co; 2-d. PubMed PMID: 11150962. 11150962

[38] Hunter SK. Sex differences in fatigability of dynamic contractions. Exp Physiol. 2016; 101(2):250–5. Epub 2015/10/07. doi: 10.1113/ep085370. PubMed PMID: 26440505; PubMed Central PMCID: PMCPMC. 26440505 PMC5777316

[39] Hunter SK. Performance Fatigability: Mechanisms and Task Specificity. Cold Spring Harb Perspect Med. 2018; 8(7). Epub 2017/05/17. doi: 10.1101/cshperspect.a029728. PubMed PMID: 28507192; PubMed Central PMCID: PMCPMC. 28507192 PMC6027928

